# Antidepressant Treatment Outcome Depends on the Quality of the Living Environment: A Pre-Clinical Investigation in Mice

**DOI:** 10.1371/journal.pone.0062226

**Published:** 2013-04-30

**Authors:** Igor Branchi, Sara Santarelli, Sara Capoccia, Silvia Poggini, Ivana D’Andrea, Francesca Cirulli, Enrico Alleva

**Affiliations:** 1 Department of Cell Biology and Neurosciences, Istituto Superiore di Sanità, Rome, Italy; 2 Institute of Anatomy, University of Zurich, Zurich, Switzerland; 3 Department of Neuro and Cardiovascular Pathology, Neuromed Insitute – Technology Park, Pozzilli, Italy; Chiba University Center for Forensic Mental Health, Japan

## Abstract

Antidepressants represent the standard treatment for major depression. However, their efficacy is variable and incomplete. A growing number of studies suggest that the environment plays a major role in determining the efficacy of these drugs, specifically of selective serotonin reuptake inhibitors (SSRI). A recent hypothesis posits that the increase in serotonin levels induced by SSRI may not affect mood *per se*, but enhances neural plasticity and, consequently, renders the individual more susceptible to the influence of the environment. Thus, SSRI administration in a favorable environment would lead to a reduction of symptoms, while in a stressful environment might lead to a worse prognosis. To test this hypothesis, we treated C57BL/6 adult male mice with chronic fluoxetine while exposing them to either (i) an enriched environment, after exposure to a chronic stress period aimed at inducing a depression-like phenotype, or (ii) a stressful environment. Anhedonia, brain BDNF and circulating corticosterone levels, considered endophenotypes of depression, were investigated. Mice treated with fluoxetine in an enriched condition improved their depression-like phenotype compared to controls, displaying higher saccharin preference, higher brain BDNF levels and reduced corticosterone levels. By contrast, when chronic fluoxetine administration occurred in a stressful condition, mice showed a more distinct worsening of the depression-like profile, displaying a faster decrease of saccharin preference, lower brain BDNF levels and increased corticosterone levels. Our findings suggest that the effect of SSRI on depression-like phenotypes in mice is not determined by the drug *per se* but is induced by the drug and driven by the environment. These findings may be helpful to explain variable effects of SSRI found in clinical practice and to device strategies aimed at enhancing their efficacy by means of controlling environmental conditions.

## Introduction

Major depression is a chronic, recurring and potentially life-threatening illness that affects up to 10% of the population across the globe. It is the leading cause of years lost owing to disability worldwide and the third overall contributor to the worldwide burden of disease (projected to be the biggest contributor by 2030) [1]. In the USA, the annual cost of depression is estimated at $ 83 billion [2] while in Europe, it is estimated over € 120 billion [3].

Antidepressants represent the current standard treatment for major depression. However, their efficacy is variable and incomplete: 60–70% of depressed patients do not experience remission and 30–40% do not show a significant response [4]. Recent publications have also cast doubts about antidepressant efficacy [5], [6], [7], claiming that when a comprehensive analysis of all trials available is performed, their effects are not significantly different from placebo [8], [9], [10], [11]. These studies have been widely criticized [12], [13] and most psychiatrists believe that antidepressants work and therefore prescribe them to patients [13]. Thus, a debate on antidepressant efficacy is open.

A new theoretical framework proposing the quality of the environment as the critical intervening factor determining the therapeutic efficacy of selective serotonin reuptake inhibitors (SSRIs) has been recently developed [14]. Such hypothesis, named the undirected susceptibility to change model, posits that the capability of the individual to change its behavior according to the environment depends on neural plasticity, which in turn is controlled by serotonin. Consequently, the increase in serotonin levels induced by SSRIs might not affect mood *per se* but enhance neural plasticity which -- acting as a catalyzer – renders the individual more susceptible to the influence of the environment. Therefore, treatment in a favorable environment, such as a high socioeconomic status [15], leads to a reduction of symptoms; by contrast, treatment in a stressful environment leads to a worse prognosis.

In support to this hypothesis, a number of indirect evidences both from clinical and preclinical studies indicate that increased serotonin levels lead to greater brain plasticity and higher susceptibility to environmental inputs [14], [16], [17], [18], [19], [20]. For instance, clinical studies investigating variations of the serotonin-transporter–linked polymorphic region, 5-HTTLPR, found that individuals bearing the s/s variant, associated with higher brain extracellular levels of serotonin, show an enhanced behavioral plasticity and susceptibility to the influence of the environment compared to individuals bearing the l/l variant [16], [21], [22]. With regard to antidepressant treatment, preliminary evidence shows that the likelihood to commit suicide is higher when patients come from a poor socioeconomic background [23]. By contrast, SSRI are more effective in patients with high socioeconomic status [4].

**Figure 1:**
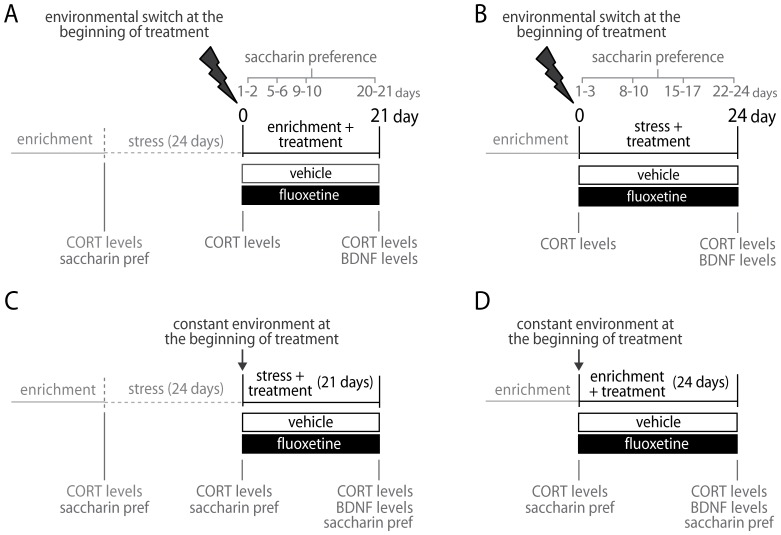
Experimental design. (A,B) Environmental switch protocols. (A) Fluoxetine treatment in an enriched condition after exposure to stress. (B) Fluoxetine treatment in a stressful condition, after exposure to enrichment. (C,D) Constant environment protocols. (C) Fluoxetine treatment in a stressful condition, after exposure to stress. (D) Fluoxetine treatment in an enriched condition, after exposure to enrichment.

The main aim of this study was to test the potential double outcome of SSRI treatment and to investigate whether it depends on the quality of the environment. To this purpose, we chronically treated mice with fluoxetine (FLX) while exposing them to either (i) an enriched condition, after a 24-days exposure to stress aimed at inducing a depression-like phenotype (Fig. 1A), or to (ii) a stressful condition, following exposure to enrichment (Fig. 1B). Mice underwent a switch in the quality of the environment to highlight its relevance in determining the effects of antidepressant administration. Three endpoints, considered endophenotypes of major depression, have been investigated: (a) anhedonia – i.e., loss of interest or pleasure in normal activities -- one of the nine symptoms defined by the *Diagnostic and Statistic Manual* (DSM-IV-TR) for major depression, which has been successfully translated in mice [24]; (b) BDNF levels, reported to be reduced in depressed patients [25], [26] and increased by antidepressant administration both in humans [27], [28] and in animal models [29], [30], [31]; (c) corticosterone levels, resulting from the activity of the hypothalamus-pituitary-adrenals axis (HPA), found to be altered in depressed patients [32], [33], [34] and in animal models [35].

In line with the undirected susceptibility to change model, our prediction was that, FLX-treated mice would be more sensitive to the quality of the environment, displaying both a better recovery from a depression-like profile, when exposed to an enriched condition, and a faster worsening when exposed to a stressful condition. In order to control for the role of the environment in driving FLX effects, further experimental groups of mice were treated while exposed to a constant environment, either enriched or stressful (Fig. 1C and 1D). In this case, we predicted that the lack of switch in the quality of the environment would lead to no modification in depression-like endpoints, with overlapping results in the FLX-treated and the control group.

## Materials and Methods

### Animals, Intellicages and enriched condition

All experiments were conducted in conformity with European Communities Council Directive 2010/63 and the Italian Decreto L.vo 116/92. The protocol was approved by the Italian Ministry of Health (Permit number 58/2012-B). All efforts were made to minimize suffering. In particular, the principles of Reduction and Refinement (i.e., the ‘three Rs’) have been applied to all experiments. C57BL/6 male mice 12–15 week old were used and kept under 12-hour light-dark cycle at 22–25 °C. Animals were housed in the Intellicage system (NewBehavior AG, Zürich, Switzerland), which is an apparatus for automatic monitoring of mouse behavior. This system is able to score the behavior of each individual living in a social group since each one is identified by a subcutaneous transponder. It consists of two large acrylic cage (20.5 cm high, 58 cm×40 cm at the top and 55 cm×37.5 cm at the base, Model 2000 Tecniplast, Buguggiate, VA, Italy), each one with 4 walls separating each corner from the center so that they form 4 identical chambers to which mice have free access by entering a front hole (for a detailed description of the system see [36], [37], [38]). Intellicages collect data about (i) number and duration of visits in the four corners (exploratory activity); (ii) number, duration and side (right or left) of nosepokes; (iii) number, duration and side (right or left) of licks.

For the entire duration of each experiment, animals were housed in two Intellicages, balancing group composition. Five days before being moved to the Intellicages, each animal was injected with a subcutaneous transponder (T-IS 8010 FDX-B; Datamars SA, Switzerland). Food was freely available. The animals have been gradually habituated to the Intellicage environment during a 16-days period. During such period, the animals were habituated also to the 0.1% of saccharin solution. On the last two days of the habituation period, baseline preference for saccharin over water in the enriched environment provided by the Intellicages was determined.

The Intellicage environment consists in an enrichment condition because mice are socially housed and are exposed to Plexiglas shelters of different colors and shapes (three red transparent Tecniplast plastic nest boxes and four white opaque boxes), and to tissue paper. New paper was provided each 5 days and the plastic shelters were cleaned and placed back each week.

### Saccharin preference

To assess saccharin preference, in each corner of the Intellicage, 2 bottles were present, one containing tap water and the other containing 0.1 % saccharin solution; both were freely available 24/24 h. Water and saccharin solution were substituted every day. The position of water and saccharin in each corner was counterbalanced across the 4 corners. Saccharin preference was determined as follows: (saccharin solution consumed/saccharin solution consumed +water consumed)×100.

### Stress condition

Stress condition consisted in exposing everyday mice to a different stressful procedure, randomly chosen among restraint, social stress or forced swim. Exposing mice to different stressful procedures was used to prevent habituation.

Restraint procedure was performed exposing animals to a 60 min restraint in a ventilated 50 ml Falcon tube (provided by hand-made holes on the tube surface). Social stress consisted in modifying social groups [36]. In particular, social group in each cage was modified so that 3 mice (2 vehicle and 1 FLX or 1 vehicle and 2 FLX) were moved from Intellicage 1 to Intellicage 2 and vice versa. This procedure forces animals to reorganize their social structure, imposing them a stressful condition. For the forced swim stress, each experimental subject was gently placed into a cylindrical glass container (20cm diameter, 40cm height), filled with 25cm of water at a temperature of 21±1°C for 10 min with a dim light illumination (1 lux). When removed from the water, the mouse was allowed to dry for 5 min under red light.

### Fluoxetine treatment

Fluoxetine (Biomol International, LP, USA) was dissolved in water and in saccharin solution and delivered *ad libitum* in the drinking bottles for 3 weeks. Compared to administration by injection, this method allows to avoid the stress due to the manipulation. The solutions were prepared according to the average weight and daily water consumption previously assessed of the mice, in order to provide an average daily intake of 10 mg/kg. The amount of water drunken by FLX-treated mice and controls was equivalent. The total amount of water and sweet solution (i.e. with saccharin) drunk by treated mice allowed to reach an effective FLX serum level around 150 ng/ml, as shown in previous studies [39]. Bottles were wrapped in tin foil to protect the substance from light. Solution was made fresh every day.

### Corticosterone levels

Corticosterone levels were measured in all subjects before and after the chronic stress procedure at baseline (i.e., no exposure to acute stress). Blood was collected from the tail 1 hr before lights on. The bleeding procedure consisted in a small and superficial cut in the tail. Blood samples were collected individually in potassium–EDTA coated 10 ml tubes (1.6 mg EDTA/ml blood; Sarstedt, Germany). All samples were kept on ice and later centrifuged at 3000 rpm for 15 min at +4°C. Blood plasma was transferred to Eppendorf tubes for corticosterone determination and stored at −20°C until further analysis. Corticosterone was measured using a commercially available radio-immunoassay (RIA) kit containing ^125^iodine labeled corticosterone (MP Biomedicals Inc., CA, USA). Vials were counted for 2 min in a gamma-scintillation counter (Packard Minaxi Gamma counter, Series 5000). Sensitivity of the assay was 0.125 mg/dl, inter- and intra-assay variation was less than 10% and 5%, respectively. Because of the asymmetric distribution, the logarithmic transformation was implemented.

### BDNF levels

Brains were collected 6 hrs after lights off and on the last day of the treatment period. The concentrations of BDNF were measured in the hippocampus and hypothalamus by an ELISA kit (BDNF Emax ImmunoAssay System number G6891, Promega, Madison, WI) according to the instructions of the manufacturer. Tissues were homogenized in the kit calibration buffer and centrifuged. The brain tissues were homogenized with ultrasonication in extraction buffer 0.2% Triton X-100. Briefly, 96-well immunoplates were coated with 100 microl per well of monoclonal anti-mouse-BDNF antibody. After an overnight incubation at 48C, the plates were washed three times with wash buffer and the samples were incubated in the coated wells (100 microl each) for 2 hr at room temperature with shaking. After an additional five washes the immobilized antigen was incubated with an antihuman BDNF antibody for 2 hr at room temperature with shaking. The plates were washed again with wash buffer, and then incubated with an anti-IgY HRP for 1 hr at room temperature. After another wash, the plates were incubated with a TMB/peroxidase substrate solution for 15 min and then phosphoric acid 1 M (100 microl/well) was added to the wells. The colorimetric reaction product was measured at 450 nm using a microplate reader (Dynatech MR 5000, PBI International, Temecula, CA). BDNF concentrations were determined, from the regression line for the BDNF standard (range ¼ 7.8– 500 pg/ml purified mouse BDNF) incubated under similar conditions in each assay. The sensitivity of the assay was about 15 pg/g of BDNF and cross-reactivity with other related neurotrophic factors (NGF, Neurotrophin-3 and Neurotrophin-4) was <3%. All assays were carried out in duplicate.

### Treatment in enriched condition, after exposure to stress

Mice underwent a 24-days stress period consisting in random exposure to different stressful conditions (i.e. restraint stress, social stress and forced swim stress). Afterwards, mice were exposed for 21 days to the enriched environment provided by the Intellicages while administered with FLX or vehicle (Fig. 1A). The preference to saccharin was monitored on days 1-3, 8-10, 15-17, 22-24 during the stress period and on days 1-2, 5-6, 9-10, 20-21 during the treatment period,. CORT levels were measured before stress exposure, immediately before drug administration (i.e. at the end of the stress exposure) and immediately after the drug administration (after 21 days). BDNF levels were measured on the last day of the treatment period.

### Treatment in stressful condition, after exposure to enrichment

After exposure to the enriched environment provided by the Intellicages, animals underwent a 24- days stress period while administered with FLX or vehicle (Fig. 1B). Saccharin preference was determined on days 1–3, 8–10, 15–17, 22–24 during the 24-days stress period. CORT levels were measured on the day before and on the last day of the treatment period. BDNF levels were measured at the end of the treatment period.

### Treatment in stressful condition, after exposure to stress

Mice underwent a 24-days stress period. Afterwards, they were exposed to a second stress period (21 days), while administered with FLX or vehicle (Fig. 1C). Preference for saccharin solution was scored on the last two days before the 24-days stress period, on the last two days of the 24-days stress period and on the last two days of the treatment period. CORT levels were measured on the day before and the last day of the 24-days stress period and at the end of the treatment period. BDNF levels were measured on the last day of the treatment period.

### Treatment in enriched condition, after exposure to enrichment

After exposure to the enriched environment provided by the Intellicages, mice have been exposed to a second enrichment period (24 days) while treated with FLX or vehicle (Fig. 1D). Preference for saccharin solution was scored on the last two days before the treatment period and on the last two days of the treatment period. CORT levels were measured at the end of the habituation period and at the end of the treatment period. BDNF levels were measured on the last day of the treatment period.

### Statistical analysis

All data were analyzed with ANOVAs, considering treatment (vehicle *vs*. FLX) as between-subject variables and subject as a random factor nested within treatment; time (day) as repeated measures within subjects. *Post hoc* comparisons were performed using the Tukey's HSD test (statistical software Statview II, Abacus Concepts, CA, USA).

## Results

### Treatment in enriched condition, after exposure to stress

#### Saccharin preference

**Figure 2:**
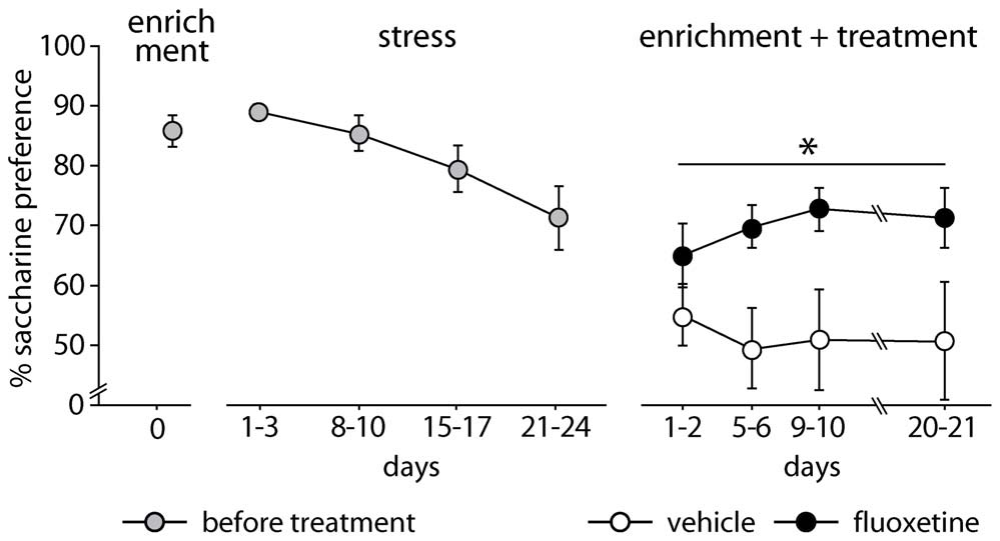
Saccharin preference during fluoxetine treatment in an enriched condition after exposure to stress. Stress exposure reduced saccharin preference in both groups of mice. Afterwards, fluoxetine-treated mice showed a significantly higher preference for the saccharin solution compared to control mice. # indicates *p* = 0.0120 and * indicates *p* = 0.0464, vs. vehicle group. Data are means ± S.E.M.

During exposure to the enrichment, the anhedonic profile was significantly affected by the treatment [F(1,14) = 4.772, p = 0.0464; n = 16 for each group]. In particular, FLX mice showed a significant preference for the saccharin solution compared to controls (Fig. 2). During the stress exposure, both groups of mice showed an overlapping significant reduction of their preference for saccharin solution [F(6,84) = 2.933, p = 0.0120]. Before the stress period, anhedonic levels were equal in the two groups.

#### Brain derived neurotrophic factor

**Figure 3:**
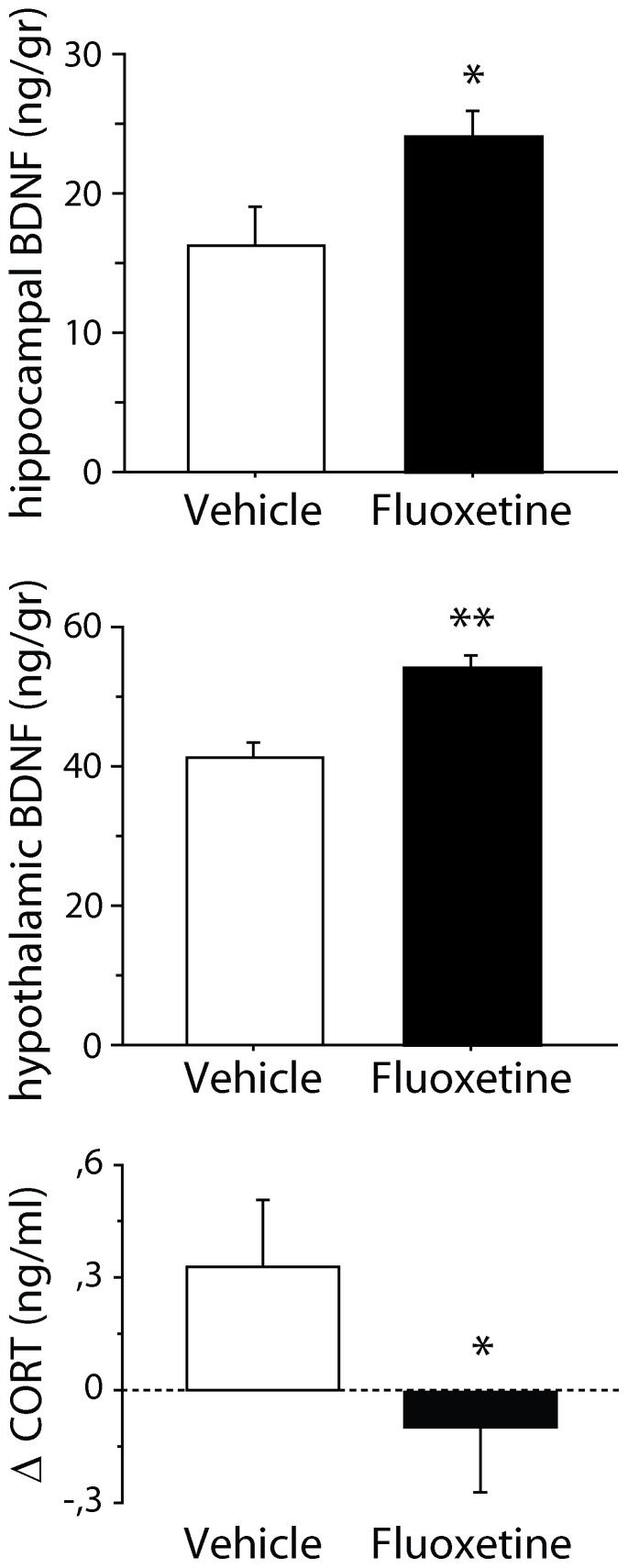
BDNF and corticosterone levels in mice in an enriched condition after exposure to stress. Following the 21 days of recovery, fluoxetine mice showed a significant increase of hippocampal and hypothalamic BDNF levels as well as significantly more marked decrease in corticosterone levels compared to controls. * and ** indicate, respectively, *p*<0.05 and 0.01 vs. vehicle group. Delta (Δ) values were calculated comparing data obtained on the day before treatment and on last day of treatment. Data are means ± S.E.M.

At the end of the treatment period, BDNF levels in the hippocampus (vehicle: n = 5; FLX: n = 5) and hypothalamus (vehicle: n = 6; FLX: n = 6) have been investigated. A significant increase in BDNF levels has been found in both brain areas in FLX mice compared to vehicle [hippocampus: F(1,8) = 5.499, p = 0.0478; hypothalamus: F(1,10) = 16.982, p = 0.0021; Fig. 3].

#### Corticosterone levels

Prior to the exposure to stress and immediately before and after treatment -- i.e., at the beginning and at the end of enriched condition –– corticosterone levels were measured (vehicle: n = 7; FLX: n = 6). When analyzing the differences between corticosterone levels before and after the 24-days stress period and before and after the treatment period, the interaction treatment x repeated measure missed statistical significance [F(1,11) = 3.941, p = 0.0726]. However, *post-hoc* analysis revealed that, during the treatment period, FLX mice displayed a significantly more marked decrease in corticosterone levels compared to vehicle (p<0.05; Fig. 3).

### Treatment in stressful condition, after exposure to enrichment

#### Saccharin preference

**Figure 4:**
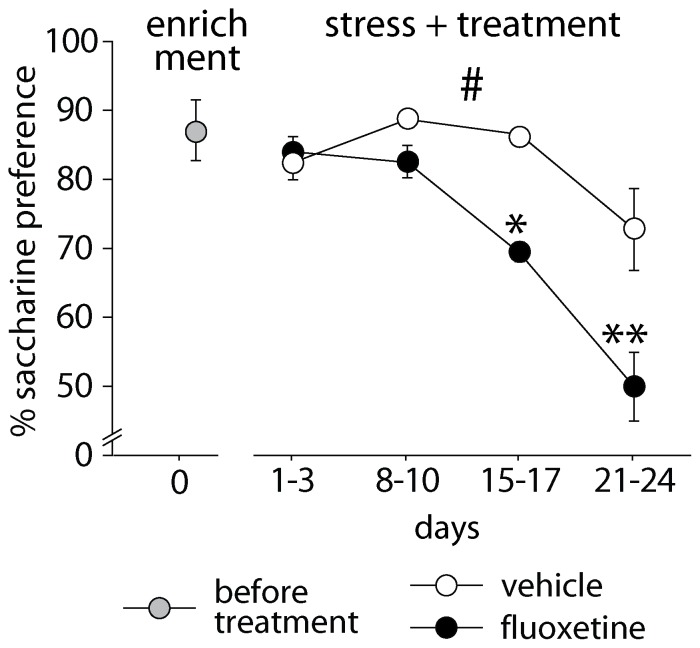
Anhedonic profile during fluoxetine treatment in a stressful condition after exposure to enrichment. When social stress was imposed during treatment, fluoxetine-treated mice showed a faster and more marked reduction of preference for the saccharin solution compared to control mice. # indicates *p* = 0.0199, * and ** indicate, respectively, *p*<0.05 and 0.01 vs. vehicle group. Data are means ± S.E.M.

During treatment in stress condition, the anhedonic profile differed in FLX mice vs. vehicle [F(1,11) = 7.410, p = 0.0199; vehicle: n = 7; FLX: n = 6]. Contrary to the enriched condition, FLX mice showed a faster and more marked reduction of preference for the saccharin solution compared to control mice (Fig. 4). In particular, the significant interaction between treatment and repeated measure [F(3,33) = 2.928, p = 0.0481] indicates that the difference between the two groups increased during the treatment, as shown by the *post-hoc* analysis (days 15-17: p<0.05; days 21-24: p<0.01). Pre-stress levels were equal in the two groups.

The effect of the combination of stress and FLX administration has been replicated in a naïve batch of animals (data not shown). Also in this case, FLX mice showed a faster and more marked reduction of saccharin preference [F(1,13) = 5.053, p = 0.0426; vehicle: n = 7; FLX: n = 8]. Though the treatment x repeated measure interaction was not significant, *post hoc* analysis revealed a significant difference between the two groups at the end of treatment (*p*<0.05).

#### Brain derived neurotrophic factor

**Figure 5:**
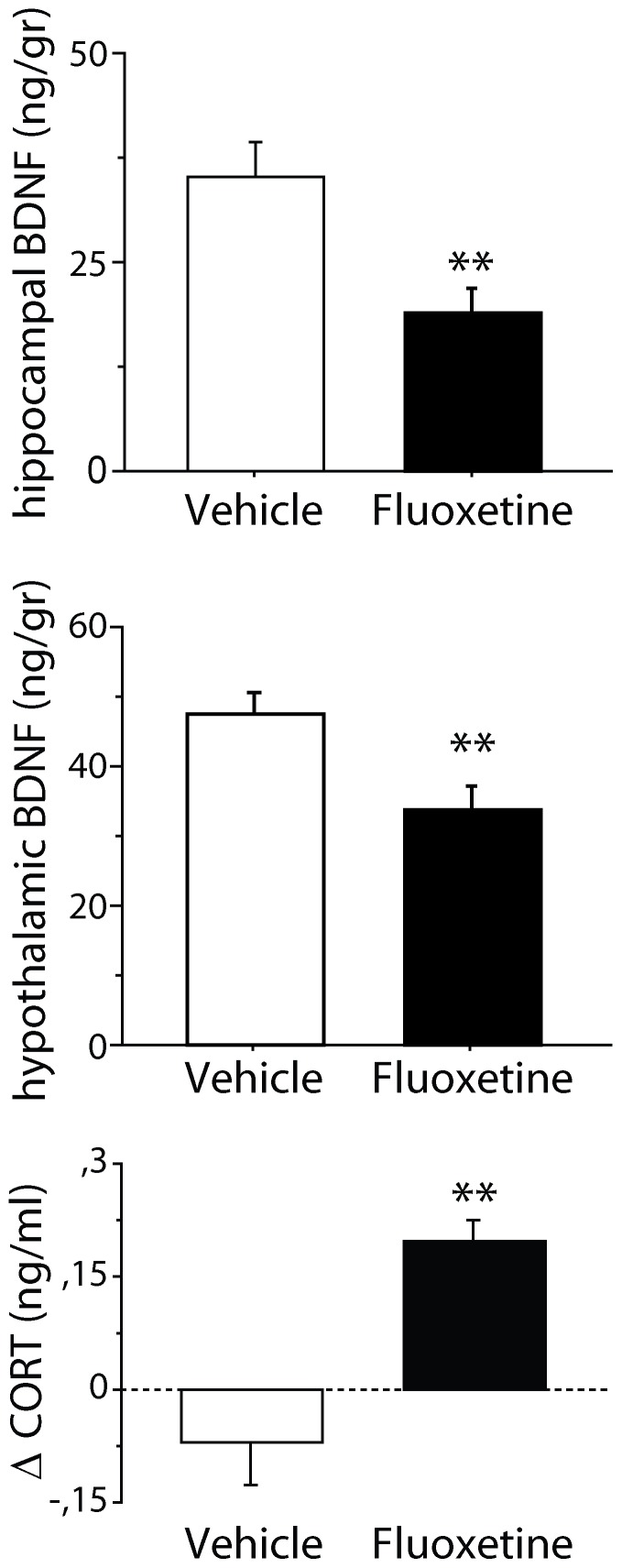
BDNF and corticosterone levels in mice in a stressful conditions after exposure to enrichment. Following social stress mice treated with fluoxetine showed reduced BDNF levels in both hippocampus and hypothalamus compared to control mice. Plasmatic corticosterone levels resulted increased in fluoxetine mice as shown by the statistically significant difference between levels before and after the treatment period. ** indicates *p*<0.01 vs. vehicle group. Delta (Δ) values were calculated comparing data obtained on the day before treatment and on last day of treatment. Data are means ± S.E.M.

At the end of the treatment period, BDNF levels in the hippocampus (vehicle: n = 6; FLX: n = 5) and hypothalamus (vehicle: n = 5; FLX: n = 4) of the experimental subjects have been investigated. A significant decrease was found in both brain areas of FLX mice compared to vehicle [hippocampus: F(1,9) = 8.846, p = 0.0156; hypothalamus: F(1,7) = 4.290, p = 0.0213; Fig. 5].

#### Corticosterone levels

At the beginning and at the end of the treatment period, corticosterone levels have been evaluated (vehicle: n = 7; FLX: n = 6). When analyzing the differences between levels before and after the treatment period, a significant increase in corticosterone levels in FLX mice compared to vehicle was found [F(1,11) = 17.018, p = 0.0017; Fig. 5].

### Treatment in stressful condition, after exposure to stress

#### Saccharin preference

**Figure 6:**
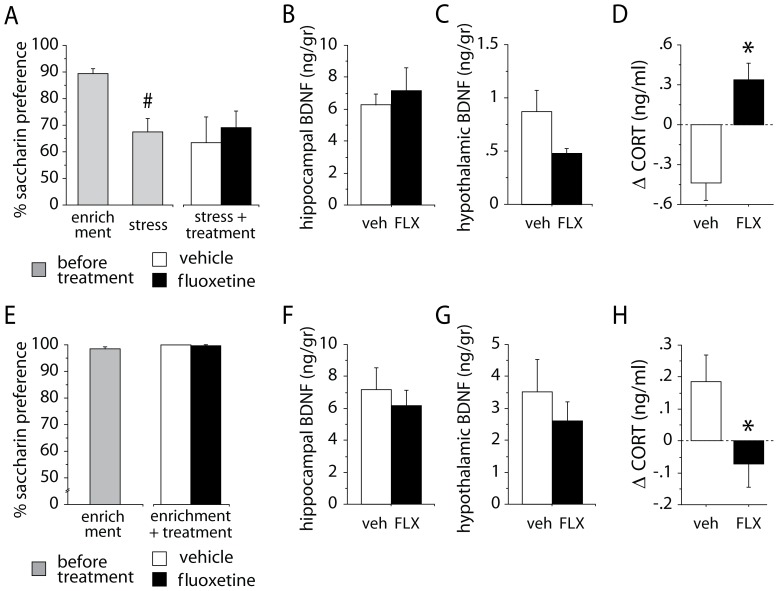
Results of experiments investigating the effects of fluoxetine treatment in a constant environment. (A,B,C,D) Fluoxetine treatment in a stressful condition, after exposure to stress. (A) Saccharin preference: exposure to stress before treatment significantly reduced saccharin preference. The following fluoxetine treatment administered in stressful conditions did not modify the anhedonic response. (B) Hippocampal and (C) hypothalamic BDNF levels: no difference between the two groups has been found. (D) Corticosterone levels. Fluoxetine mice showed a significant increase in corticosterone levels compared to vehicle. (E,F,G,H) Fluoxetine treatment in an enriched condition, after exposure to enrichment. (E) Saccharin preference: no difference between the two groups has been found. (F) Hippocampal and (G) hypothalamic BDNF levels: no difference between the two groups has been found. (H) Corticosterone levels: fluoxetine mice showed a significant decrease in corticosterone levels compared to vehicle. * indicates *p*<0.05 vs. vehicle group. # indicates *p*<0.01 vs. baseline level. Delta (Δ) values were calculated comparing data obtained on the day before treatment and on last day of treatment. Data are means ± S.E.M.

At the end of the treatment period, FLX and control mice did not differ in saccharin preference [F(1,34) = 0.225, p = 0.6383; vehicle: n = 19; FLX: n = 17]. Both groups showed a preference around 60–70%. Preference levels were significantly reduced by exposure to the 24-days stress period [F(1,34) = 15.653, p = 0.0004; Fig. 6A].

#### Brain derived neurotrophic factor

BDNF levels in the hippocampus and hypothalamus (vehicle: n = 11; FLX: n = 8) have been investigated at the end of the treatment period. No difference in BDNF levels between the two groups was found in the two brain areas [respectively, Fs(1,17) = 0.429, 2.845, ps = 0.5213, 0.1099; Fig 6B and 6C].

#### Corticosterone levels

The analysis performed on the differences in corticosterone levels measured before and after the exposure to the 24-day stress period and before and after the treatment period showed a significant treatment x repeated measure interaction [F(1,14) = 6.548, p = 0.0227; vehicle: n = 7; FLX: n = 9]. *Post-hoc* analysis revealed that, during the treatment period, FLX mice displayed a significant increase in corticosterone levels compared to vehicle (p<0.05; Fig. 6D).

### Treatment in enriched condition, after exposure to enrichment

#### Saccharin preference

At the end of treatment in enriched conditions, the anhedonic profile of FLX and control mice did not differ [F(1,11) = 0.002, p = 0.9642; vehicle: n = 11; FLX: n = 11]. Both groups showed a very high preference for the saccharin solution, around 100% (Fig. 6E).

#### Brain derived neurotrophic factor

BDNF levels in the hippocampus (vehicle: n = 8; FLX: n = 11) and hypothalamus (vehicle: n = 8; FLX: n = 11) have been investigated at the end of the treatment period. No difference in BDNF levels has been found in both brain areas of FLX mice compared to vehicle [respectively: Fs(1,17) = 0.386, ps = 0.5428, 0.4326; Fig. 6F and 6G].

#### Corticosterone levels

The analysis of the differences in corticosterone levels before and at the end of treatment revealed a significant treatment x repeated measure interaction [F(1,14) = 17.989, p = 0.008; vehicle: n = 7; FLX: n = 9]. *Post-hoc* analysis showed that, during the treatment period, FLX mice displayed a significant decrease in corticosterone levels compared to vehicle (p<0.05; Fig. 6H).

## Discussion

Our results show that, after a period of stress, which induces a depression-like phenotype, mice exposed to a favorable environment, such as enrichment, recovered when treated with FLX, displaying reduced anhedonia, higher brain BDNF levels and lower corticosterone levels compared to controls. By contrast, when chronic FLX administration occurred in a stressful condition, mice showed a more pronounced worsening of behavioral and neuroendocrine endpoints indicative of a depression-like phenotype. Specifically, FLX-treated mice showed a faster decrease of saccharin preference, already evident after two weeks of treatment, and had lower brain BDNF levels and increased corticosterone levels. When treatment was administered in a constant environment, FLX did not affect the depression-like phenotype and the two experimental groups showed overlapping results, with the exception of corticosterone levels. Concerning the latter parameter, FLX increased levels of this hormone in a stressful condition, while reducing it in an enriched environment.

Results concerning the effects of FLX in an enriched condition, after exposure to stress, are in line with the expected effects of antidepressants [40]. Indeed, treatment allowed a recovery from the depression-like phenotype, improving the three endpoints considered symptoms/biomarkers of the psychopathology. However, these effects cannot be ascribed only to drug action *per se*, but are due to the combination of treatment and the environmental context. In fact, different results are obtained when the antidepressant is administered in a different environmental context. In particular, results indicate that a switch from an enriched to a stressful environment increases anhedonia in control mice, in line with the literature [41], [42] and that FLX amplifies such effect. The latter finding, replicated twice in the present study, is not in line with some previous data indicating an increased preference for the sweet solution following SSRI administration [43], [44]. Nonetheless, other data in the literature show increased anhedonia following SSRI administration [45], [46], [47], [48]. A similar picture has been reported for the effects of SSRIs administered in a stressful condition on BDNF and corticosterone levels. Indeed, the reduction of BDNF levels here reported is incongruent with some studies showing that SSRIs increase levels of this neurotrophin [29], [43]. However, other studies found that chronic FLX has no effect [49], [50], [51] or even reduces BDNF levels [52], [53], [54], [55], [56], [57]. With regard to HPA axis activity, though some studies described a reduction of corticosterone levels [58], [59], mainly mediated by an increased expression of glucocorticoid receptors [60], others have indicated opposite effects [61], [62]. The inverted relationship between corticosterone and BDNF levels found in mice exposed to either the enriched or the stressful condition has already been reported [63], [64], [65]. In addition, the association between the low BDNF levels and the anhedonic profile, indicative of a depression-like phenotype, is in line with studies in patients [26], [27], [28]. The lack of FLX effects on saccharin preference and BDNF levels in constant environmental conditions confirms that effects of the drug are driven by the environment. The significant change in corticosterone levels, even in a constant environment, on the one hand suggests that this parameter is more sensitive than anhedonia and BDNF to the effects of the combination of the drug and the environment, being altered even after a period of habituation, on the other, confirms that FLX treatment amplifies the effects of the environment, either supportive or adverse. Overall, the results here described are in line and support the undirected susceptibility to change model positing that an enhancement of the serotonin system increases the individual’s plasticity and, thus, the sensitivity to the environmental context [14].

Though very few studies on patients have investigated the influence of the environment on antidepressant action, their findings show that living conditions, e.g. socioeconomic status, modulate the effects of antidepressants. In line with the present results, low-income people who suffer from depression are less likely to respond to antidepressant treatment than those in middle- and high-income groups [23], [66]. In addition, findings from the Sequenced Treatment Alternatives to Relieve Depression (STAR*D) study, which investigated the response to the SSRI citalopram in over 4000 depressed patients, showed that participants who were Caucasian, female, employed or had higher levels of education or income had higher remission rates. By contrast, longer index episodes, more concurrent psychiatric disorders, more general medical disorders and lower baseline function and quality of life were associated with lower remission rates [4]. The relevance of social inequalities in affecting antidepressant treatment outcome has been highlighted by the World Health Organization [67].

Recently, doubts have been brought about on antidepressant efficacy [5], [6], [7], [8]. In particular, the studies by Irvin Kirsch showed that, compared to placebos, different antidepressants have a very limited effect, possibly of no clinical significance. In addition, Kirsch speculated that even such a limited effect might be an artifact due to the fact that side effects enable patients to guess that they are receiving the active drug, making them more likely to report an improvement in symptoms [11]. In line with this hypothesis, in trials employing “active” placebos causing side, no differential effects with drug treatment were found [10]. However, most psychiatrists, based on their own clinical experience, believe that antidepressants work and thus prescribe them to depressed patients [12], [13], [68]. Indeed, antidepressants are among the most prescribed drugs in western countries [69].

Data from this study provide an explanation for the variability found in clinical trials [70], [71] and for the fact that an effective treatment may result ineffective. In clinical trials the environment in which patients live is not -- or is only partially – controlled. As a consequence, the proportion of patients living in a supportive or, vice versa, in a stressful environment is likely to change from trial to trial: when such proportions are approximately equal, an absence of effect would be reported, because the beneficial and harmful effects of SSRI treatment may compensate each other; otherwise, when the number of patients exposed to a supportive or a stressful environment is not balanced, a positive or negative treatment outcome may be found. The undirected susceptibility to change model also justifies the lack of a difference in the effects between high and low doses [8] and explains other incongruities about SSRI effects reported in the literature, such as the high placebo response rate and the efficacy of treatments based on opposite mechanisms of action (e.g., SSRI vs. serotonin selective reuptake enhancers; for a detailed description, see [14]).

Some limitations in our interpretation of the experimental results need to be underlined. First, part of the assumptions is mainly based on results collected using animal studies. Therefore, *ad hoc* clinical and epidemiological studies are needed to further test the validity of our hypothesis. In addition, the serotonergic system was considered as acting as a whole, not taking into account its high molecular complexity and its wide range of effects, such as those on food intake or circadian rhythm. Finally, further studies involving experimental groups exposed to a standard, neither enriched nor stressful, environment are warranted to better describe the interplay between treatment and quality of the environment.

In conclusion, our findings suggest that SSRI administration does have an effect. However, such effect is not determined by the drug *per se* but is induced by the drug and driven by the environment. Such critical role of the environment is corroborated by our results showing that FLX treatment has limited effects when administered in constant environmental conditions. This view may be helpful to better understand SSRI effects and selectively enhance their efficacy through the control of environmental conditions in patients. This could be achieved by training patients to cope with harsh environments, for instance through cognitive behavioral therapy [72], since it is unlikely that people can rapidly and effectively change their living milieu. The cost of this approach is limited since no new psychoactive molecules need to be developed, while the benefits for the patients could be substantial.

## References

[1] WHO (2008) The global burden of disease: 2004 update: World Health Organization.

[2] Greenberg PE, Kessler RC, Birnbaum HG, Leong SA, Lowe SW, et al (2003) The economic burden of depression in the United States: how did it change between 1990 and 2000? J Clin Psychiatry 64: 1465–1475. 14728109 10.4088/jcp.v64n1211

[3] Balak N, Elmaci I (2007) Costs of disorders of the brain in Europe. Eur J Neurol 14: e9. 10.1111/j.1468-1331.2006.01570.x 17250716

[4] Trivedi MH, Rush AJ, Wisniewski SR, Nierenberg AA, Warden D, et al (2006) Evaluation of outcomes with citalopram for depression using measurement-based care in STAR*D: implications for clinical practice. Am J Psychiatry 163: 28–40. 16390886 10.1176/appi.ajp.163.1.28

[5] Angell M (2008) Industry-sponsored clinical research: a broken system. JAMA 300: 1069–1071. 18768418 10.1001/jama.300.9.1069

[6] Fournier JC, DeRubeis RJ, Hollon SD, Dimidjian S, Amsterdam JD, et al (2010) Antidepressant drug effects and depression severity: a patient-level meta-analysis. JAMA 303: 47–53. 20051569 10.1001/jama.2009.1943 PMC3712503

[7] Turner EH, Matthews AM, Linardatos E, Tell RA, Rosenthal R (2008) Selective publication of antidepressant trials and its influence on apparent efficacy. N Engl J Med 358: 252–260. 18199864 10.1056/NEJMsa065779

[8] Kirsch I, Deacon BJ, Huedo-Medina TB, Scoboria A, Moore TJ, et al (2008) Initial severity and antidepressant benefits: a meta-analysis of data submitted to the Food and Drug Administration. PLoS Med 5: e45. 18303940 10.1371/journal.pmed.0050045 PMC2253608

[9] Moncrieff J, Kirsch I (2005) Efficacy of antidepressants in adults. BMJ 331: 155–157. 16020858 10.1136/bmj.331.7509.155 PMC558707

[10] Moncrieff J, Wessely S, Hardy R (2005) Active Placebos Versus Antidepressants for Depression. The Cochrane Library 4 10.1002/14651858.CD003012.pub2 PMC8407353 14974002

[11] Kirsch I (2010) The Emperor's New Drugs: Exploding the Antidepressant Myth. New York: Basic Books.

[12] Fountoulakis KN, Moller HJ (2011) Efficacy of antidepressants: a re-analysis and re-interpretation of the Kirsch data. Int J Neuropsychopharmacol 14: 405–412. 20800012 10.1017/S1461145710000957

[13] McAllister-Williams RH (2008) Do antidepressants work? A commentary on "Initial severity and antidepressant benefits: a meta-analysis of data submitted to the Food and Drug Administration" by Kirsch et al. Evid Based Ment Health 11: 66–68. 18669671 10.1136/ebmh.11.3.66

[14] Branchi I (2011) The double edged sword of neural plasticity: increasing serotonin levels leads to both greater vulnerability to depression and improved capacity to recover. Psychoneuroendocrinology 36: 339–351. 20875703 10.1016/j.psyneuen.2010.08.011

[15] Hackman DA, Farah MJ, Meaney MJ (2010) Socioeconomic status and the brain: mechanistic insights from human and animal research. Nat Rev Neurosci 11: 651–659. 20725096 10.1038/nrn2897 PMC2950073

[16] Belsky J, Jonassaint C, Pluess M, Stanton M, Brummett B, et al (2009) Vulnerability genes or plasticity genes? Mol Psychiatry 14: 746–754. 19455150 10.1038/mp.2009.44 PMC2834322

[17] Homberg JR (2012) Genetic sensitivity to the environment, across lifetime. Behav Brain Sci 35: 368. 10.1017/S0140525X1200101X 23095389

[18] Kiser D, Steemers B, Branchi I, Homberg JR (2011) The reciprocal interaction between serotonin and social behaviour. Neurosci Biobehav Rev 10.1016/j.neubiorev.2011.12.009 22206901

[19] Maya Vetencourt JF, Sale A, Viegi A, Baroncelli L, De Pasquale R, et al (2008) The antidepressant fluoxetine restores plasticity in the adult visual cortex. Science 320: 385–388. 18420937 10.1126/science.1150516

[20] Karpova NN, Pickenhagen A, Lindholm J, Tiraboschi E, Kulesskaya N, et al (2011) Fear erasure in mice requires synergy between antidepressant drugs and extinction training. Science 334: 1731–1734. 22194582 10.1126/science.1214592 PMC3929964

[21] Brummett BH, Boyle SH, Siegler IC, Kuhn CM, Ashley-Koch A, et al (2008) Effects of environmental stress and gender on associations among symptoms of depression and the serotonin transporter gene linked polymorphic region (5-HTTLPR). Behav Genet 38: 34–43. 17955359 10.1007/s10519-007-9172-1 PMC2777886

[22] Eley TC, Sugden K, Corsico A, Gregory AM, Sham P, et al (2004) Gene-environment interaction analysis of serotonin system markers with adolescent depression. Mol Psychiatry 9: 908–915. 15241435 10.1038/sj.mp.4001546

[23] Cohen A, Houck PR, Szanto K, Dew MA, Gilman SE, et al (2006) Social inequalities in response to antidepressant treatment in older adults. Arch Gen Psychiatry 63: 50–56. 16389196 10.1001/archpsyc.63.1.50

[24] Cryan JF, Holmes A (2005) The ascent of mouse: advances in modelling human depression and anxiety. Nat Rev Drug Discov 4: 775–790. 16138108 10.1038/nrd1825

[25] Cirulli F, Alleva E (2009) The NGF saga: from animal models of psychosocial stress to stress-related psychopathology. Front Neuroendocrinol 30: 379–395. 19442684 10.1016/j.yfrne.2009.05.002

[26] Sen S, Duman R, Sanacora G (2008) Serum brain-derived neurotrophic factor, depression, and antidepressant medications: meta-analyses and implications. Biol Psychiatry 64: 527–532. 18571629 10.1016/j.biopsych.2008.05.005 PMC2597158

[27] Hellweg R, Ziegenhorn A, Heuser I, Deuschle M (2008) Serum concentrations of nerve growth factor and brain-derived neurotrophic factor in depressed patients before and after antidepressant treatment. Pharmacopsychiatry 41: 66–71. 18311687 10.1055/s-2007-1004594

[28] Shimizu E, Hashimoto K, Okamura N, Koike K, Komatsu N, et al (2003) Alterations of serum levels of brain-derived neurotrophic factor (BDNF) in depressed patients with or without antidepressants. Biol Psychiatry 54: 70–75. 12842310 10.1016/s0006-3223(03)00181-1

[29] Nibuya M, Nestler EJ, Duman RS (1996) Chronic antidepressant administration increases the expression of cAMP response element binding protein (CREB) in rat hippocampus. J Neurosci 16: 2365–2372. 8601816 10.1523/JNEUROSCI.16-07-02365.1996 PMC6578518

[30] Chourbaji S, Brandwein C, Gass P (2011) Altering BDNF expression by genetics and/or environment: impact for emotional and depression-like behaviour in laboratory mice. Neurosci Biobehav Rev 35: 599–611. 20621121 10.1016/j.neubiorev.2010.07.003

[31] Chourbaji S, Hortnagl H, Molteni R, Riva MA, Gass P, et al (2012) The impact of environmental enrichment on sex-specific neurochemical circuitries - effects on brain-derived neurotrophic factor and the serotonergic system. Neuroscience 220: 267–276. 22710068 10.1016/j.neuroscience.2012.06.016

[32] Nemeroff CB, Widerlov E, Bissette G, Walleus H, Karlsson I, et al (1984) Elevated concentrations of CSF corticotropin-releasing factor-like immunoreactivity in depressed patients. Science 226: 1342–1344. 6334362 10.1126/science.6334362

[33] Holsboer F (2000) The corticosteroid receptor hypothesis of depression. Neuropsychopharmacology 23: 477–501. 11027914 10.1016/S0893-133X(00)00159-7

[34] Ising M, Horstmann S, Kloiber S, Lucae S, Binder EB, et al (2007) Combined dexamethasone/corticotropin releasing hormone test predicts treatment response in major depression - a potential biomarker? Biol Psychiatry 62: 47–54. 17123470 10.1016/j.biopsych.2006.07.039

[35] Chourbaji S, Brandwein C, Vogt MA, Dormann C, Gass P (2008) Evaluation of effects of previous exposure to an acute stressor before testing for depression-like behaviours in mice. Stress 11: 170–175. 18311605 10.1080/10253890701560119

[36] Branchi I, D'Andrea I, Cirulli F, Lipp HP, Alleva E (2010) Shaping brain development: mouse communal nesting blunts adult neuroendocrine and behavioral response to social stress and modifies chronic antidepressant treatment outcome. Psychoneuroendocrinology 35: 743–751. 19945226 10.1016/j.psyneuen.2009.10.016

[37] Knapska E, Walasek G, Nikolaev E, Neuhausser-Wespy F, Lipp HP, et al (2006) Differential involvement of the central amygdala in appetitive versus aversive learning. Learn Mem 13: 192–200. 16547163 10.1101/lm.54706 PMC1409843

[38] Mechan AO, Wyss A, Rieger H, Mohajeri MH (2009) A comparison of learning and memory characteristics of young and middle-aged wild-type mice in the IntelliCage. J Neurosci Methods 180: 43–51. 19427528 10.1016/j.jneumeth.2009.02.018

[39] Dulawa SC, Holick KA, Gundersen B, Hen R (2004) Effects of chronic fluoxetine in animal models of anxiety and depression. Neuropsychopharmacology 29: 1321–1330. 15085085 10.1038/sj.npp.1300433

[40] Kramer PD (1994) Listening to Prozac: Psychiatrist Explores Antidepressant Drugs and the Remaking of the Self London: Fourth Estate Ltd.

[41] Willner P (1997) Validity, reliability and utility of the chronic mild stress model of depression: a 10-year review and evaluation. Psychopharmacology (Berl) 134: 319–329. 10.1007/s002130050456 9452163

[42] Strekalova T, Spanagel R, Bartsch D, Henn FA, Gass P (2004) Stress-induced anhedonia in mice is associated with deficits in forced swimming and exploration. Neuropsychopharmacology 29: 2007–2017. 15266352 10.1038/sj.npp.1300532

[43] Bessa JM, Ferreira D, Melo I, Marques F, Cerqueira JJ, et al (2009) The mood-improving actions of antidepressants do not depend on neurogenesis but are associated with neuronal remodeling. Mol Psychiatry 14: 764–773, 739. 18982002 10.1038/mp.2008.119

[44] Rygula R, Abumaria N, Flugge G, Hiemke C, Fuchs E, et al (2006) Citalopram counteracts depressive-like symptoms evoked by chronic social stress in rats. Behav Pharmacol 17: 19–29. 16377960 10.1097/01.fbp.0000186631.53851.71

[45] Brenes JC, Fornaguera J (2009) The effect of chronic fluoxetine on social isolation-induced changes on sucrose consumption, immobility behavior, and on serotonin and dopamine function in hippocampus and ventral striatum. Behav Brain Res 198: 199–205. 19027796 10.1016/j.bbr.2008.10.036

[46] Prendergast MA, Yells DP, Balogh SE, Paige SR, Hendricks SE (2002) Fluoxetine differentially suppresses sucrose solution consumption in free-fed and food-deprived rats--reversal by amantadine. Med Sci Monit 8: BR385–390. 12388910

[47] Sammut S, Bethus I, Goodall G, Muscat R (2002) Antidepressant reversal of interferon-alpha-induced anhedonia. Physiol Behav 75: 765–772. 12020742 10.1016/s0031-9384(02)00677-7

[48] Tonissaar M, Mallo T, Eller M, Haidkind R, Koiv K, et al (2008) Rat behavior after chronic variable stress and partial lesioning of 5-HT-ergic neurotransmission: effects of citalopram. Prog Neuropsychopharmacol Biol Psychiatry 32: 164–177. 17826880 10.1016/j.pnpbp.2007.08.001

[49] Conti AC, Cryan JF, Dalvi A, Lucki I, Blendy JA (2002) cAMP response element-binding protein is essential for the upregulation of brain-derived neurotrophic factor transcription, but not the behavioral or endocrine responses to antidepressant drugs. J Neurosci 22: 3262–3268. 11943827 10.1523/JNEUROSCI.22-08-03262.2002 PMC6757540

[50] Dias BG, Banerjee SB, Duman RS, Vaidya VA (2003) Differential regulation of brain derived neurotrophic factor transcripts by antidepressant treatments in the adult rat brain. Neuropharmacology 45: 553–563. 12907316 10.1016/s0028-3908(03)00198-9

[51] Altieri M, Marini F, Arban R, Vitulli G, Jansson BO (2004) Expression analysis of brain-derived neurotrophic factor (BDNF) mRNA isoforms after chronic and acute antidepressant treatment. Brain Res 1000: 148–155. 15053962 10.1016/j.brainres.2003.12.028

[52] Jacobsen JP, Mork A (2004) The effect of escitalopram, desipramine, electroconvulsive seizures and lithium on brain-derived neurotrophic factor mRNA and protein expression in the rat brain and the correlation to 5-HT and 5-HIAA levels. Brain Res 1024: 183–192. 15451381 10.1016/j.brainres.2004.07.065

[53] Kozisek ME, Middlemas D, Bylund DB (2008) Brain-derived neurotrophic factor and its receptor tropomyosin-related kinase B in the mechanism of action of antidepressant therapies. Pharmacol Ther 117: 30–51. 17949819 10.1016/j.pharmthera.2007.07.001

[54] Miro X, Perez-Torres S, Artigas F, Puigdomenech P, Palacios JM, et al (2002) Regulation of cAMP phosphodiesterase mRNAs expression in rat brain by acute and chronic fluoxetine treatment. An in situ hybridization study. Neuropharmacology 43: 1148–1157. 12504921 10.1016/s0028-3908(02)00220-4

[55] Zetterstrom TS, Pei Q, Madhav TR, Coppell AL, Lewis L, et al (1999) Manipulations of brain 5-HT levels affect gene expression for BDNF in rat brain. Neuropharmacology 38: 1063–1073. 10428425 10.1016/s0028-3908(99)00022-2

[56] Alboni S, Benatti C, Capone G, Corsini D, Caggia F, et al (2010) Time-dependent effects of escitalopram on brain derived neurotrophic factor (BDNF) and neuroplasticity related targets in the central nervous system of rats. Eur J Pharmacol 643: 180–187. 20599917 10.1016/j.ejphar.2010.06.028

[57] Goekint M, Roelands B, Heyman E, Njemini R, Meeusen R (2011) Influence of citalopram and environmental temperature on exercise-induced changes in BDNF. Neurosci Lett 494: 150–154. 21385602 10.1016/j.neulet.2011.03.001

[58] Pariante CM, Thomas SA, Lovestone S, Makoff A, Kerwin RW (2004) Do antidepressants regulate how cortisol affects the brain? Psychoneuroendocrinology 29: 423–447. 14749091 10.1016/j.psyneuen.2003.10.009

[59] Uys JD, Muller CJ, Marais L, Harvey BH, Stein DJ, et al (2006) Early life trauma decreases glucocorticoid receptors in rat dentate gyrus upon adult re-stress: reversal by escitalopram. Neuroscience 137: 619–625. 16310967 10.1016/j.neuroscience.2005.08.089

[60] Anacker C, Zunszain PA, Carvalho LA, Pariante CM (2011) The glucocorticoid receptor: pivot of depression and of antidepressant treatment? Psychoneuroendocrinology 36: 415–425. 20399565 10.1016/j.psyneuen.2010.03.007 PMC3513407

[61] Shen Q, Lal R, Luellen BA, Earnheart JC, Andrews AM, et al (2010) gamma-Aminobutyric acid-type A receptor deficits cause hypothalamic-pituitary-adrenal axis hyperactivity and antidepressant drug sensitivity reminiscent of melancholic forms of depression. Biol Psychiatry 68: 512–520. 20579975 10.1016/j.biopsych.2010.04.024 PMC2930197

[62] Weber CC, Eckert GP, Muller WE (2006) Effects of antidepressants on the brain/plasma distribution of corticosterone. Neuropsychopharmacology 31: 2443–2448. 16641944 10.1038/sj.npp.1301076

[63] Berry A, Bellisario V, Capoccia S, Tirassa P, Calza A, et al (2012) Social deprivation stress is a triggering factor for the emergence of anxiety- and depression-like behaviours and leads to reduced brain BDNF levels in C57BL/6J mice. Psychoneuroendocrinology 37: 762–772. 21974975 10.1016/j.psyneuen.2011.09.007

[64] Jacobsen JP, Mork A (2006) Chronic corticosterone decreases brain-derived neurotrophic factor (BDNF) mRNA and protein in the hippocampus, but not in the frontal cortex, of the rat. Brain Res 1110: 221–225. 16876769 10.1016/j.brainres.2006.06.077

[65] Ridder S, Chourbaji S, Hellweg R, Urani A, Zacher C, et al (2005) Mice with genetically altered glucocorticoid receptor expression show altered sensitivity for stress-induced depressive reactions. J Neurosci 25: 6243–6250. 15987954 10.1523/JNEUROSCI.0736-05.2005 PMC6725059

[66] Cohen A, Gilman SE, Houck PR, Szanto K, Reynolds CF 3rd (2009) Socioeconomic status and anxiety as predictors of antidepressant treatment response and suicidal ideation in older adults. Soc Psychiatry Psychiatr Epidemiol 44: 272–277. 18818858 10.1007/s00127-008-0436-8 PMC2662042

[67] WHO (2010) Equity, social determinants and public health programmes. 10.1111/j.1600-0528.2011.00623.x 21623864

[68] Feifel D (2008) More depressing news on antidepressants: should we panic? Psychiatry (Edgmont) 5: 35–36. PMC2719549 19727307

[69] Olfson M, Marcus SC (2009) National patterns in antidepressant medication treatment. Arch Gen Psychiatry 66: 848–856. 19652124 10.1001/archgenpsychiatry.2009.81

[70] Kirsch I, Scoboria A, Moore TJ (2002) Antidepressants and placebos: secrets, revelations, and unanswered questions. Prev Treat 5: article 33.

[71] Moncrieff J (2003) A comparison of antidepressant trials using active and inert placebos. Int J Methods Psychiatr Res 12: 117–127. 12953139 10.1002/mpr.148 PMC6878450

[72] Wiles N, Thomas L, Abel A, Ridgway N, Turner N et al.. (2012) Cognitive behavioural therapy as an adjunct to pharmacotherapy for primary care based patients with treatment resistant depression: results of the CoBalT randomised controlled trial. Lancet. 10.1016/S0140-6736(12)61552-9 23219570

